# Oral Supplementation with a Kudzu Root–Black Soybean Blend Attenuates Ovarian Dysfunction in Mice: Implications for Its Potential Use as a Dietary Supplement in Animal Nutrition

**DOI:** 10.3390/ani16162591

**Published:** 2026-08-19

**Authors:** Tongtong Song, Guoda A, Jialing He, Changyong Zhang, Nan Li, Tianqi Wang, Yuan Xu, Guo Bao

**Affiliations:** 1 College of Animal Science and Technology, Northeast Agricultural University, Harbin 150030, China; songtt010220@163.com (T.S.); aguoda02@gmail.com (G.A.); 2 Laboratory Animal Center, National Research Institute for Family Planning, Beijing 100081, China; missu329@aliyun.com (J.H.); zhang789752@163.com (C.Z.); linan@nrifp.org.cn (N.L.); 2003wangtianqi@163.com (T.W.)

**Keywords:** *Pueraria lobata*, *Glycine max*, dietary feed additive, ovarian dysfunction, reproductive performance, oxidative stress

## Abstract

Kudzu (*Pueraria lobata*) root and black soybean (*Glycine max*) are plant-derived feed ingredients rich in bioactive phytochemicals, including puerarin, isoflavones, and anthocyanins. These compounds are recognized for their antioxidant and physiological regulatory properties, supporting their potential as dietary components in livestock nutrition. In this study, we evaluated the effects of the Pueraria–black soybean compound powder (KRBS) as a dietary additive on female reproductive performance using a cyclophosphamide (CTX)-induced ovarian dysfunction mouse model. The results showed that the high-dose KRBS group (CTX+H-KRBS) had a significantly increased ovarian index (*p* < 0.05), and all KRBS-treated groups recovered estrous cycle regularity (*p* < 0.01). Molecular analysis showed that KRBS significantly upregulated *Amh* expression (*p* < 0.05), decreased *Bax* expression, and increased *Bcl2* expression, while reducing *Esr1* and *Vegfa* expression in uterine tissues to levels comparable to those in the control group (*p* < 0.05). These molecular changes suggest that KRBS may alleviate CTX-induced ovarian dysfunction through the regulation of apoptosis-related gene expression, including *Bax* and *Bcl2*. These findings indicate that KRBS supplementation alleviates CTX-induced ovarian dysfunction through improvements in follicular development, reduction in serum FSH toward control levels, and apoptosis-related gene expression. These effects support the potential application of KRBS as a plant-based dietary supplement for preserving ovarian function.

## Simple Summary

Environmental stress, poor nutrition, and oxidative damage frequently impair reproductive performance in female livestock, resulting in substantial economic losses. In this study, we evaluated whether a natural plant-based feed supplement derived from Kudzu (*Pueraria lobata*) root and black soybean (*Glycine max*) could alleviate ovarian dysfunction. The Kudzu–black soybean formulation was given as a dietary supplement to mice with impaired ovarian function. Dietary supplementation significantly improved ovarian tissue architecture, promoted normal follicular development, and reduced follicular atresia. Treated mice also showed a recovery in circulating reproductive hormone levels. Reproductive outcomes showed favorable trends but did not reach statistical significance. At the cellular level, the supplement attenuated apoptosis in ovarian tissue, suggesting a protective effect on the ovarian reserve. These findings suggest that the Kudzu–black soybean formulation may support ovarian function and endocrine regulation, warranting further evaluation in livestock.

## 1. Introduction

Female reproductive performance is a major determinant of productivity and sustainability in livestock production systems [1,2]. Ovarian function is essential for regulating follicular development, endocrine homeostasis, and overall fertility [3]. However, environmental stress, nutritional imbalance, aging, and exposure to toxic compounds can compromise ovarian function in livestock, leading to disrupted folliculogenesis, endocrine disorders, and impaired reproductive performance [4,5]. Consequently, developing safe nutritional strategies to preserve ovarian function is an important objective in animal production [6]. In traditional Chinese veterinary medicine, botanical ingredients with complementary biological activities are commonly combined to improve animal health and reproductive performance, providing a potential basis for developing phytogenic feed additives [7]. This complementary approach may be particularly relevant for reproductive disorders involving multiple interacting biological processes [8].

Phytogenic feed ingredients have attracted increasing attention in animal nutrition due to their diverse biological activities and favorable safety profiles [9,10]. Among botanical candidates, *Pueraria lobata* (kudzu) is rich in puerarin and other isoflavonoids. Previous studies have demonstrated that *P. lobata* exhibits antioxidant, anti-inflammatory, and endocrine-modulating activities [11,12], indicating a potential role in supporting ovarian function. Black soybean (*Glycine max*) has a long history of dietary and medicinal use and is abundant in isoflavones, anthocyanins, and polyphenols. Previous studies have also shown that black soybean-derived phytochemicals alleviate oxidative stress and improve endocrine homeostasis [13,14], supporting their potential relevance to ovarian function. The complementary biological activities of these two botanicals provide a rationale for their combined use.

Oxidative stress is a major contributor to ovarian dysfunction [15], as excessive accumulation of reactive oxygen species (ROS) can induce granulosa cell apoptosis and follicular atresia, ultimately impairing reproductive performance [16]. *P. lobata* provides puerarin and related isoflavones with antioxidant and phytoestrogenic activities, while black soybean contributes additional isoflavones, anthocyanins, and polyphenols that may support antioxidant defense and endocrine regulation. Collectively, this evidence provides a basis for evaluating the effects of KRBS on ovarian dysfunction. Consistent with this reasoning, a fermented formulation combining *P. lobata* with *Glycine max* extracts has been reported to ameliorate dyslipidemia and metabolic abnormalities in ovariectomized mice [17]. Although that study used conventional soybean-derived extracts rather than black soybean specifically, it provides supporting evidence that combining Pueraria with soybean-derived ingredients can produce complementary physiological benefits in a hormone-deficient model.

However, whether a Pueraria–black soybean formulation can protect against chemotherapy-induced ovarian injury remains unknown. In this study, we evaluated a Pueraria–black soybean compound powder (KRBS) as a dietary supplement using a cyclophosphamide (CTX)-induced mouse model of ovarian dysfunction. Ovarian morphology, follicular development, endocrine function, reproductive performance, and apoptosis-related signaling pathways were examined. The results of this study provide experimental support for the potential use of KRBS as a phytogenic feed additive for preserving ovarian function.

## 2. Materials and Methods

### 2.1. Animals and Ethical Statements

Female and male Kunming mice (specific-pathogen-free (SPF) grade, 9 weeks old) were purchased in two batches from Vital River Laboratory Animal Technology Co., Ltd. (SCXK (Beijing) 2021-0006, Beijing, China). All animals were housed at the Experimental Animal Center of the National Institute of Health Science (SYXK (Beijing) 2025-0043) under SPF conditions.

Female mice were used for ovarian dysfunction model establishment and dietary intervention, whereas male mice were used for mating and fertility evaluation. Animals were maintained under a 12 h light/12 h dark cycle at 21 ± 2 °C and 50–60% relative humidity, with free access to food and water.

All experimental procedures were approved by the Animal Welfare and Ethics Committee of the National Institute of Health Science (Approval No. NRIFH21-2403-20).

### 2.2. Experimental Design, Cyclophosphamide Induction, and Dietary Supplementation with KRBS

Fifty female Kunming mice with similar body weights were randomly assigned to five groups (*n* = 10 per group, based on previous CTX-induced ovarian dysfunction studies): normal control (CON, treated with an equal volume of normal saline), cyclophosphamide-treated control group (CTX), low-dose Pueraria–black soybean compound group (CTX+L-KRBS), medium-dose Pueraria–black soybean compound group (CTX+M-KRBS), and high-dose Pueraria–black soybean compound group (CTX+H-KRBS). Except for the CON group, mice in the other four groups were injected intraperitoneally (i.p.) with cyclophosphamide (CTX) to induce ovarian dysfunction. Before injection, mice were weighed, and CTX was administered at a dose of 80 mg/kg once daily for 14 consecutive days [18,19]. The CTX working solution was prepared in sterile physiological saline at a concentration of 0.80 mg/mL. The injection volume for each mouse was calculated according to body weight using the following formula:

Injection Volume (mL) = [80 mg/kg × Body weight (kg)]/0.80 mg/mL

Throughout the experimental period, body weight was recorded weekly, and the general health status and mortality of the animals were monitored daily. On day 14 of CTX administration, vaginal smears were collected from randomly assigned mice to evaluate estrous cycle alterations and confirm ovarian dysfunction induction.

After completion of the cyclophosphamide induction period, all mice continued to receive the basal diet provided to the CON group, and an 8-week oral gavage intervention was initiated. KRBS was commercially supplied as a plant-derived compound powder formulated from *Pueraria lobata* root and *Glycine max* (black soybean). The product was supplied in dry powder form and stored in sealed containers under conditions at room temperature until use. The chemical characterization and nutritional composition of KRBS are presented in Table 1. Before oral gavage, KRBS was dispersed in purified water at the required concentrations and thoroughly mixed to prepare homogeneous suspensions. Based on the human equivalent intake of 2 g/day for a 60 kg adult, the corresponding doses of KRBS for mice were calculated and set at 100, 200, and 400 mg·kg^−1^·day^−1^ for the low-, medium-, and high-dose groups, respectively. To ensure a standardized gavage volume of 500 μL per 20 g body weight, the KRBS suspension concentrations for the low-, medium-, and high-dose groups were adjusted to 12, 24, and 48 mg/mL, respectively. The daily gavage volume for each mouse was calculated according to the following formula:

Dose × 1000 × body weight (kg)/concentration (mg/mL)

During the 8-week intervention period, mice in the CON and CTX groups received an equal volume of purified water by oral gavage daily, whereas mice in the CTX+L-KRBS, CTX+M-KRBS, and CTX+H-KRBS groups received corresponding volumes of KRBS suspension. All groups received oral gavage once daily at a fixed time for 8 consecutive weeks.

### 2.3. Estrous Cycle Analysis

Female Kunming mice were restrained, and after exposure of the vaginal opening, a sterile cotton swab moistened with sterile saline was inserted into the vagina to a depth of approximately 3–5 mm and rotated to collect vaginal secretions. The swab was then rolled onto a glass slide to prepare a smear, which was allowed to air-dry. Following Giemsa staining, cellular morphology was examined under a microscope. The estrous cycle stage was determined based on the predominant cell types: a predominance of nucleated epithelial cells indicated proestrus; a predominance of anucleated cornified cells indicated estrus; a mixture of leukocytes and epithelial cells indicated metestrus; and a predominance of leukocytes indicated diestrus. Throughout the procedure, the insertion depth was controlled to avoid injury.

### 2.4. Mating Behavior and Fertility Assessment

Mating experiments were conducted by co-housing female and male mice at a female-to-male ratio of 2:1 starting at 17:00 daily. The following morning at 8:00, female mice were examined for vaginal plugs, and the day of plug detection was designated as gestational day 0.5. This mating procedure was continued for 5 consecutive days, with vaginal plug examination performed daily. Females with vaginal plugs were housed individually for gestation and delivery. The delivery date was recorded to calculate gestation duration, and offspring-related parameters were collected. Within 24 h postpartum, litter size (including stillbirths) was recorded, individual pup body weights were measured, and average litter weight was calculated. At weaning (postnatal day 21), the number of surviving pups per litter was recorded to calculate the weaning survival rate (number of surviving pups/litter size × 100%), and individual pup body weights were measured to calculate average litter weight at weaning.

### 2.5. Tissue Collection and Histopathology

At the end of the experiment, animals were euthanized by cervical dislocation after 6 h of fasting with free access to water. Ovaries and uteri were collected and weighed. The ovarian index and uterine index were determined as organ-to-body weight ratios. Subsequently, tissues were fixed in 4% paraformaldehyde at 4 °C for 24–48 h.

Fixed tissues were dehydrated, cleared, and embedded in paraffin. Ovarian tissues were serially sectioned to a thickness of 5 μm, and one section was selected from every five consecutive sections for analysis. Follicles were classified and quantified as primordial, primary, secondary, antral, and atretic follicles under a microscope.

### 2.6. Hormone Measurement

Serum levels of estradiol (E2), luteinizing hormone (LH), and follicle-stimulating hormone (FSH) were measured using commercially available enzyme-linked immunosorbent assay (ELISA) kits (Wuhan Elabscience Biotechnology Co., Ltd., Wuhan, China). The assay procedure included sample loading, incubation at 37 °C, washing, TMB substrate development, reaction termination, and absorbance measurement at 450 nm.

### 2.7. RNA Extraction and Reverse Transcription

Total RNA was extracted from the ovarian and uterine tissues of mice using the TRIzol reagent. The concentration and purity of the isolated RNA were determined using a NanoDrop 2000 spectrophotometer (Thermo Fisher Scientific, Waltham, MA, USA). Subsequently, 1 μg of total RNA was reverse-transcribed into complementary DNA (cDNA) using the PrimeScript RT Reagent Kit with gDNA Eraser (Takara, Shiga, Japan).

### 2.8. Quantitative Real-Time PCR (qPCR)

Real-time PCR was performed on a StepOnePlus Real-Time PCR System (Thermo Fisher Scientific, Waltham, MA, USA) using TB Green Premix Ex Taq (Takara, Shiga, Japan). The primer sequences used in this study are provided in Appendix A. The PCR reaction mixture (total volume of 20 μL) contained 10 μL of TB Green Premix Ex Taq, 0.8 μL of forward primer (10 μM), 0.8 μL of reverse primer (10 μM), 2.0 μL of cDNA template, and 6.4 μL of nuclease-free water. *Actb* (β-actin) was used as the internal reference gene. The relative expression levels of the target genes were calculated using the comparative 2^−ΔΔCt^ method. All reactions were performed in triplicate.

### 2.9. Statistical Analysis

All statistical analyses were performed using SPSS 19.0 software (IBM Corp, Armonk, NY, USA). Data are presented as mean ± standard deviation (SD). Repeated measures analysis of variance (ANOVA) was used for longitudinal comparisons. One-way ANOVA followed by the least significant difference (LSD) post hoc test was used for comparisons among multiple groups. A *p*-value < 0.05 was considered statistically significant.

## 3. Results

### 3.1. Effects of KRBS on Body Weight and Estrous Cyclicity in Mice

**Figure 1:**
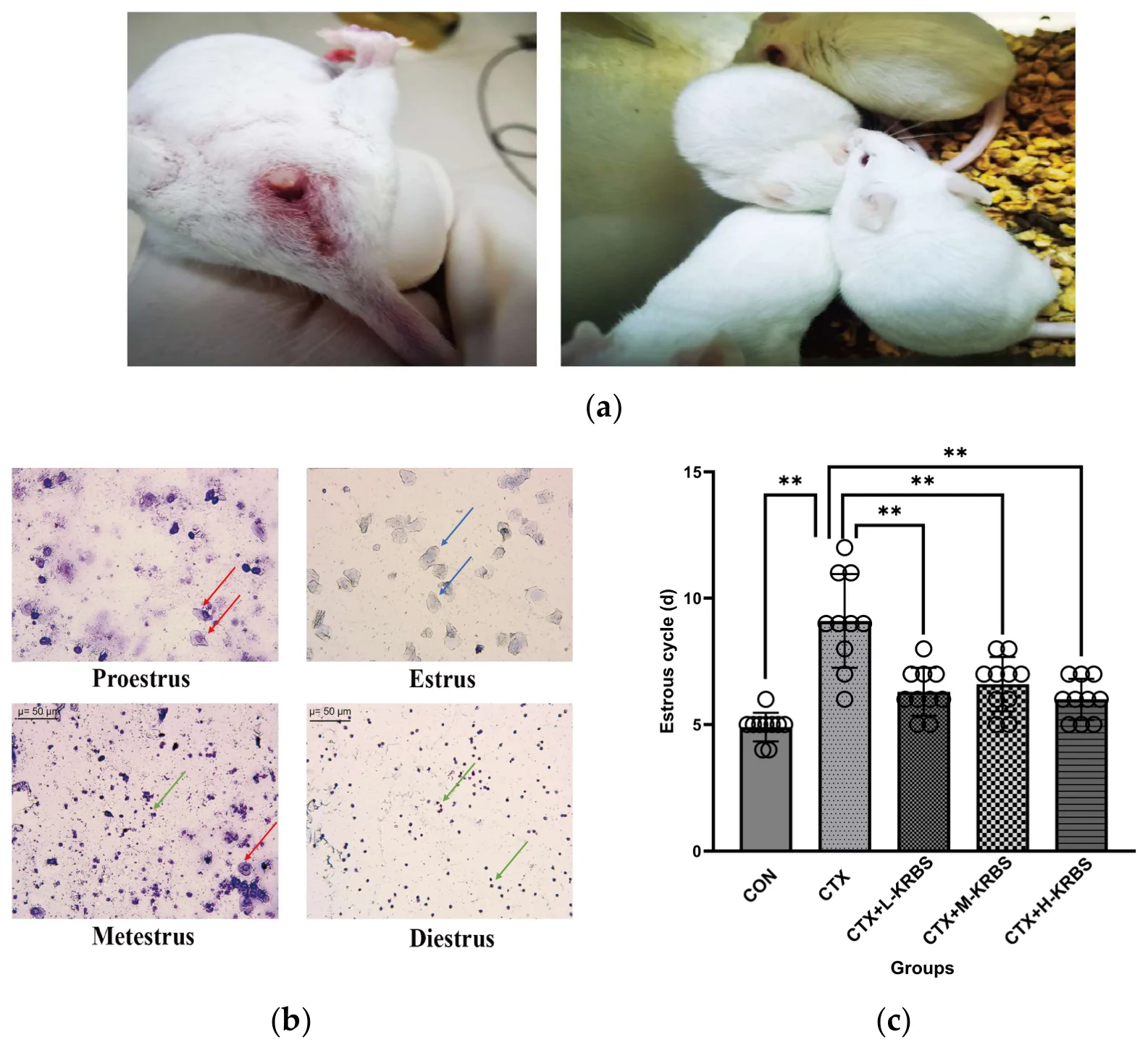
Effects of KRBS supplementation on general condition and estrous cyclicity in CTX-induced ovarian dysfunction mice. (**a**) Representative photographs of general condition during the CTX administration period. (**b**) Representative vaginal cytology images at different estrous stages. Red arrows, nucleated epithelial cells; blue arrows, cornified squamous epithelial cells; green arrows, leukocytes. Scale bar = 50 μm. (**c**) Estrous cycle duration. Data are presented as mean ± SD (*n* = 10 per group). ** *p* < 0.01 vs. the CTX group. CON, normal control; CTX, cyclophosphamide; KRBS, Pueraria–black soybean compound powder; L-KRBS, low-dose KRBS; M-KRBS, medium-dose KRBS; H-KRBS, high-dose KRBS.

During the CTX administration period, mice displayed signs of discomfort, including huddling, pale ear margins, and ruffled fur (Figure 1a). CTX-treated mice showed a trend toward lower body weight relative to the CON group, although this difference did not reach statistical significance. Over the 8-week KRBS intervention period, body weight remained numerically higher in the CON group, with no significant differences between the CTX+KRBS groups and the CTX group. Notably, KRBS-treated mice gained weight progressively across the experimental period, indicating that KRBS supplementation did not adversely affect body weight (Table 2).

Before CTX administration, all mice had regular estrous cycles (approximately 5–6 days). Estrous cycle stages were determined by vaginal cytology based on predominant cell types: nucleated epithelial cells indicated proestrus; cornified epithelial cells indicated estrus; a mixture of epithelial cells and leukocytes indicated metestrus; and predominant leukocytes indicated diestrus (Figure 1b). Following CTX administration, the CTX group showed marked disruption of the estrous cycle, characterized by prolonged diestrus and reduced occurrence of estrus and proestrus phases, consistent with CTX-induced ovarian dysfunction. Estrous cycle length was significantly prolonged in the CTX group compared with the CON group (Figure 1c). Conversely, KRBS supplementation at all doses (CTX+L-KRBS, CTX+M-KRBS, and CTX+H-KRBS) significantly shortened estrous cycle duration, restoring it toward normal levels (*p* < 0.01). These results indicate that KRBS supplementation alleviates estrous cycle disruption caused by CTX-induced ovarian injury.

### 3.2. Effects of KRBS on Ovarian Morphology and Follicular Dynamics

**Figure 2:**
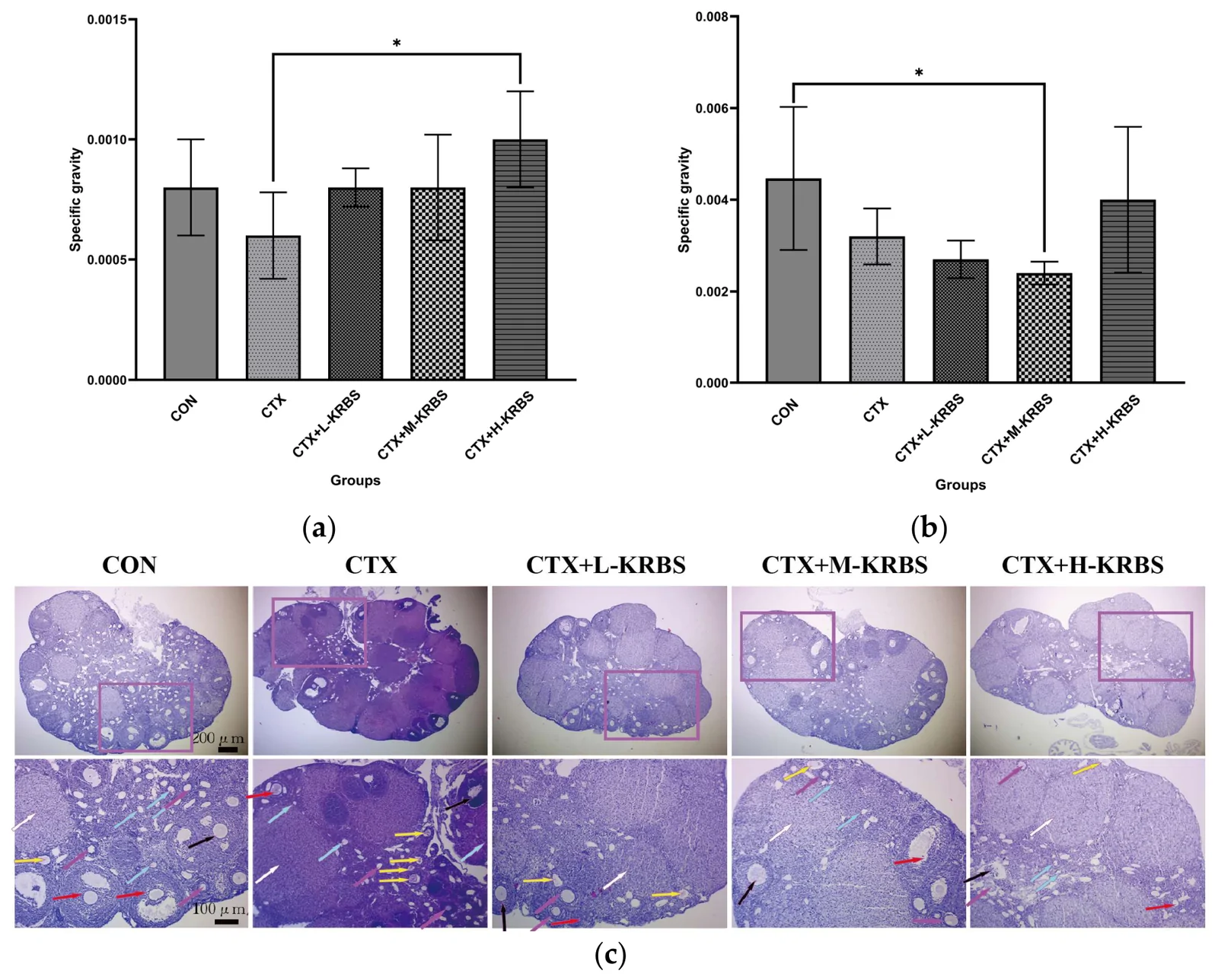

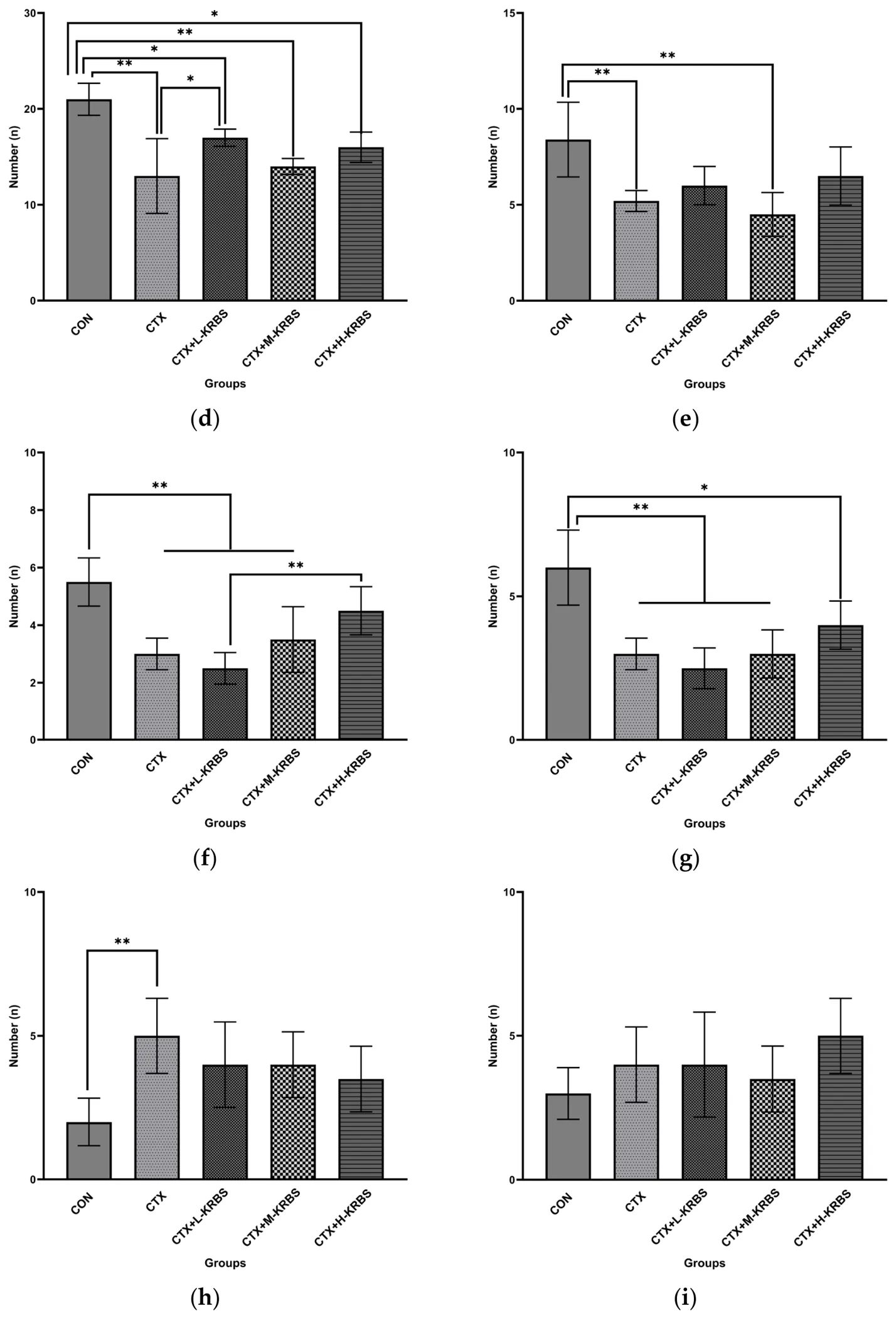
Effects of KRBS supplementation on ovarian morphology and follicular development in CTX-induced ovarian dysfunction mice. (**a**) Relative weight of the ovaries. (**b**) Relative weight of the uterus. (**c**) Representative hematoxylin and eosin (HE)-stained ovarian sections. Arrows indicate follicle types: blue, primordial follicles; purple, primary follicles; black, secondary follicles; red, antral follicles; yellow, atretic follicles. (**d**–**i**) Quantification of follicle populations: (**d**) primordial, (**e**) primary, (**f**) secondary, (**g**) antral, and (**h**) atretic follicles; (**i**) corpora lutea. Data are presented as mean ± SD (n = 10 per group). * *p* < 0.05, ** *p* < 0.01 vs. the CON group or the CTX group, as stated in the main text. CON, normal control; CTX, cyclophosphamide; KRBS, Pueraria–black soybean compound powder; L-KRBS, low-dose KRBS; M-KRBS, medium-dose KRBS; H-KRBS, high-dose KRBS.

Following CTX administration, the CTX group showed a trend toward a lower ovarian index compared with the CON group, although this difference was not statistically significant (Figure 2a). This reduction was attenuated by KRBS supplementation. Ovarian indices in the CTX+L-KRBS and CTX+M-KRBS groups approached the levels observed in the CON group, while the CTX+H-KRBS group had a significantly higher ovarian index than the CTX group (*p* < 0.05). For the uterine index (Figure 2b), the CTX group showed a trend toward a lower value compared with the CON group, although this difference was not statistically significant. The CTX+M-KRBS group showed a further significant reduction compared with the CON group (*p* < 0.05). In contrast, the CTX+H-KRBS group showed partial restoration of uterine index, approaching the level observed in the CON group.

Histological analysis (Figure 2c) revealed a higher number of atretic follicles in the CTX group than in the CON group, whereas the CTX+M-KRBS and CTX+H-KRBS groups showed a trend toward fewer atretic follicles and more primordial follicles. Quantitative analysis of follicular dynamics (Figure 2d) confirmed that CTX administration significantly reduced primordial follicle numbers relative to the CON group (*p* < 0.01). KRBS supplementation was associated with an increasing trend in primordial follicle numbers, although only the CTX+L-KRBS group reached statistical significance versus the CTX group (*p* < 0.05); no significant differences were detected among the three KRBS dosage groups for this parameter.

Further quantitative analysis showed that the CON group had significantly higher numbers of primordial, primary, secondary, and antral follicles compared with the CTX group (*p* < 0.01). Among KRBS-treated groups, primordial follicle numbers were significantly elevated in the CTX+L-KRBS group relative to the CTX group (*p* < 0.05). For secondary follicles, the CTX+H-KRBS group had significantly higher counts than the CTX+L-KRBS group (*p* < 0.01). No significant differences were observed among KRBS doses for the remaining follicular parameters. Antral follicle numbers were significantly higher in the CON group than in the CTX, CTX+L-KRBS, and CTX+M-KRBS groups (*p* < 0.01), and the difference between the CON and CTX+H-KRBS groups remained significant (*p* < 0.05). The CTX group displayed a significant increase in atretic follicle numbers compared with the CON group (*p* < 0.01); KRBS-treated groups showed only a trend toward fewer atretic follicles, without reaching statistical significance (*p* > 0.05). Corpora lutea numbers did not differ significantly among groups (*p* > 0.05).

These results suggest that KRBS supplementation alleviates CTX-induced ovarian damage by preserving primordial follicle populations and promoting follicular development, accompanied by a partial improvement in uterine status.

### 3.3. Effects of KRBS on Serum Hormone Levels

**Figure 3:**
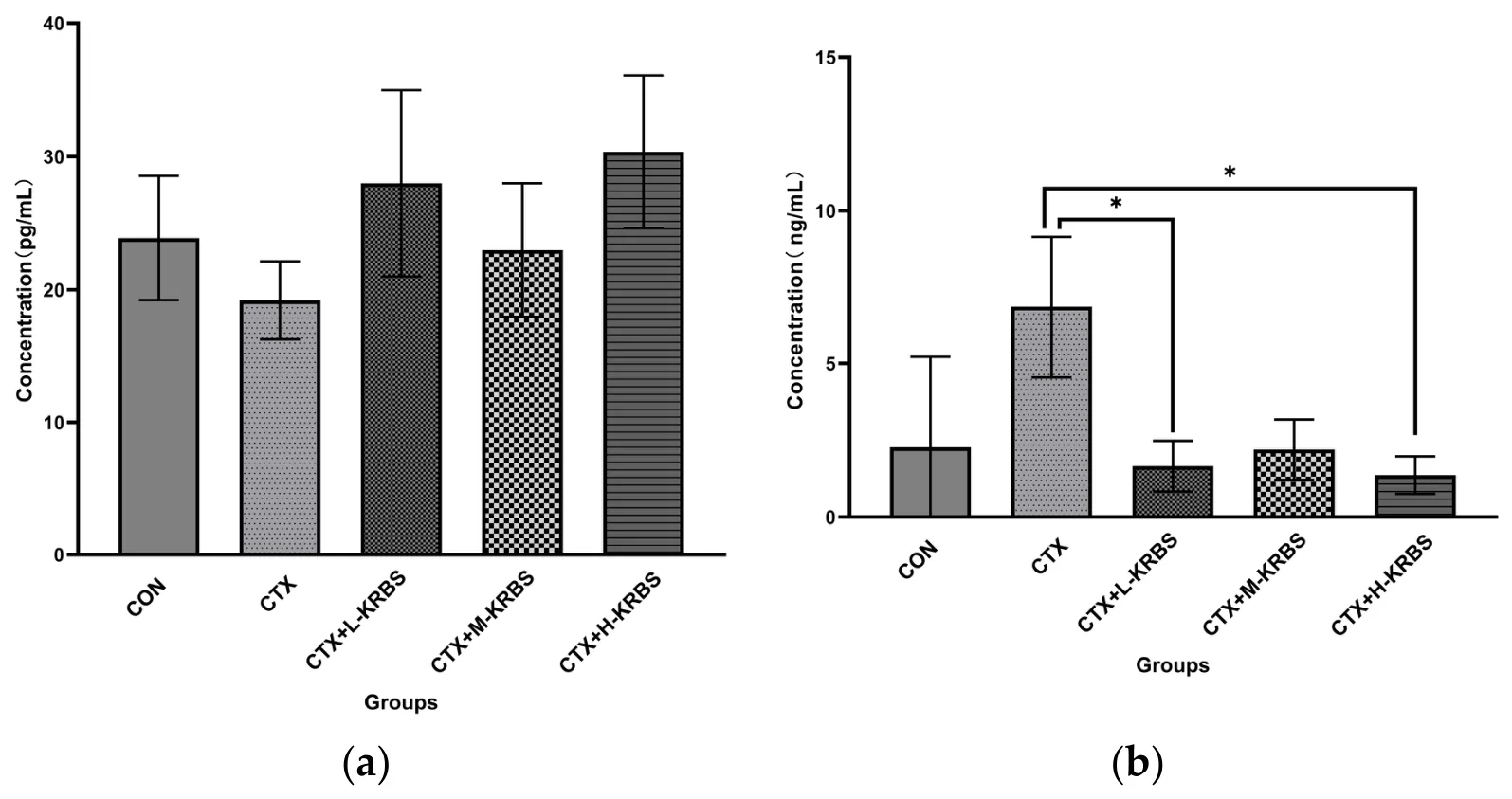

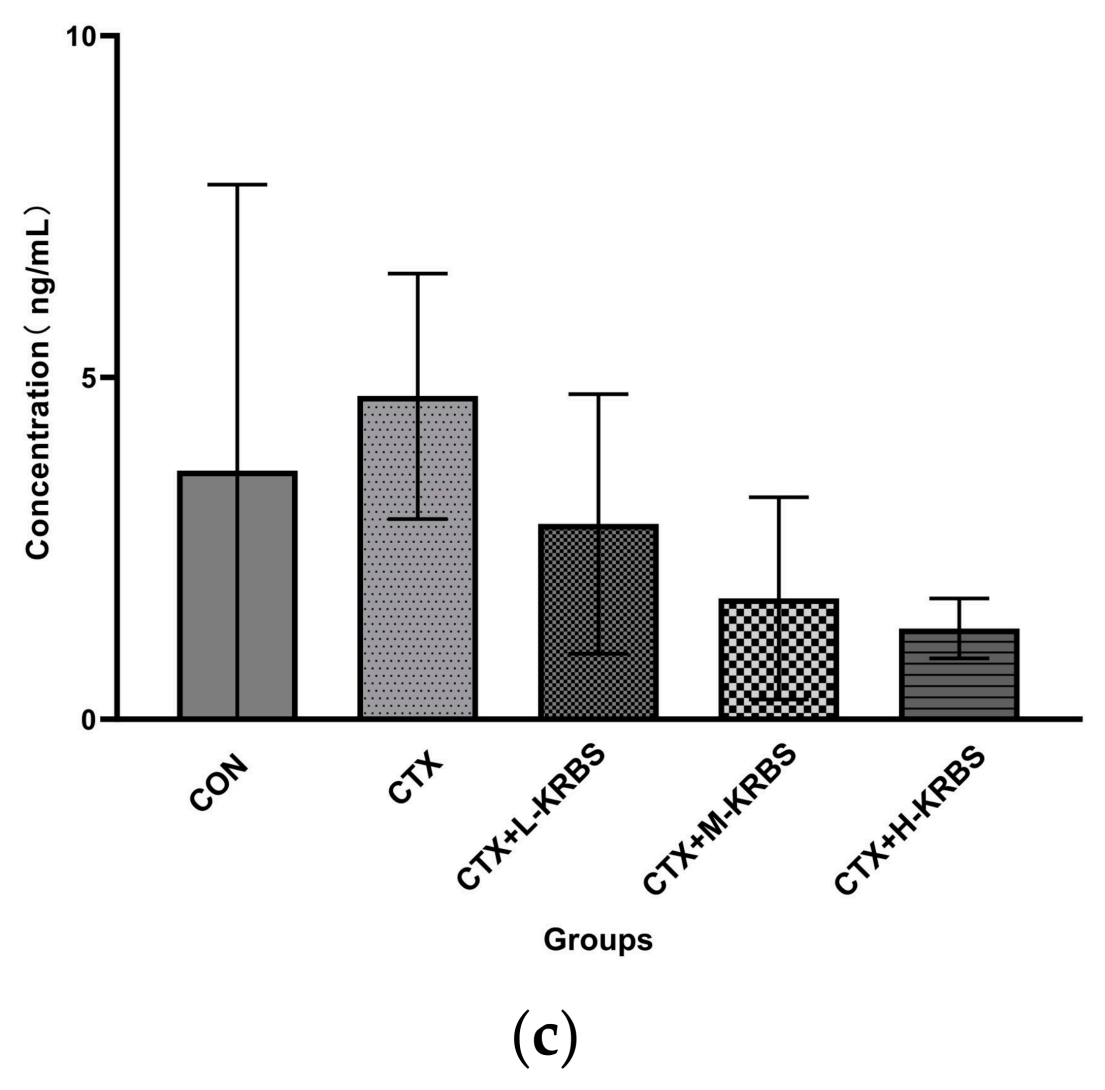
Effects of KRBS supplementation on serum levels of E2 (**a**), FSH (**b**), and LH (**c**) in CTX-induced ovarian dysfunction mice. Data are presented as mean ± SD (*n* = 10 per group). * *p* < 0.05 vs. the CTX group. CON, normal control; CTX, cyclophosphamide; KRBS, Pueraria–black soybean compound powder; L-KRBS, low-dose KRBS; M-KRBS, medium-dose KRBS; H-KRBS, high-dose KRBS; E2, estradiol; FSH, follicle-stimulating hormone; LH, luteinizing hormone.

Hypothalamic–pituitary–ovarian (HPO) axis function was evaluated by measuring serum levels of E2, FSH, and LH (Figure 3). Serum E2 levels trended lower in the CTX group than in the CON group, but this difference was not statistically significant (Figure 3a). FSH also trended higher in the CTX group than in the CON group, without reaching statistical significance. Compared with the CTX group, FSH levels were significantly lower in the CTX+L-KRBS and CTX+H-KRBS groups (*p* < 0.05) (Figure 3b). LH levels trended downward in all KRBS-supplemented groups (CTX+L-KRBS, CTX+M-KRBS, and CTX+H-KRBS) compared with the CTX group, although none reached statistical significance (Figure 3c). KRBS supplementation was associated with partial improvement in serum hormone levels in mice with CTX-induced ovarian dysfunction, particularly reflected by the reduction in FSH toward control levels.

### 3.4. Effects of KRBS on Reproductive Outcomes

**Figure 4:**
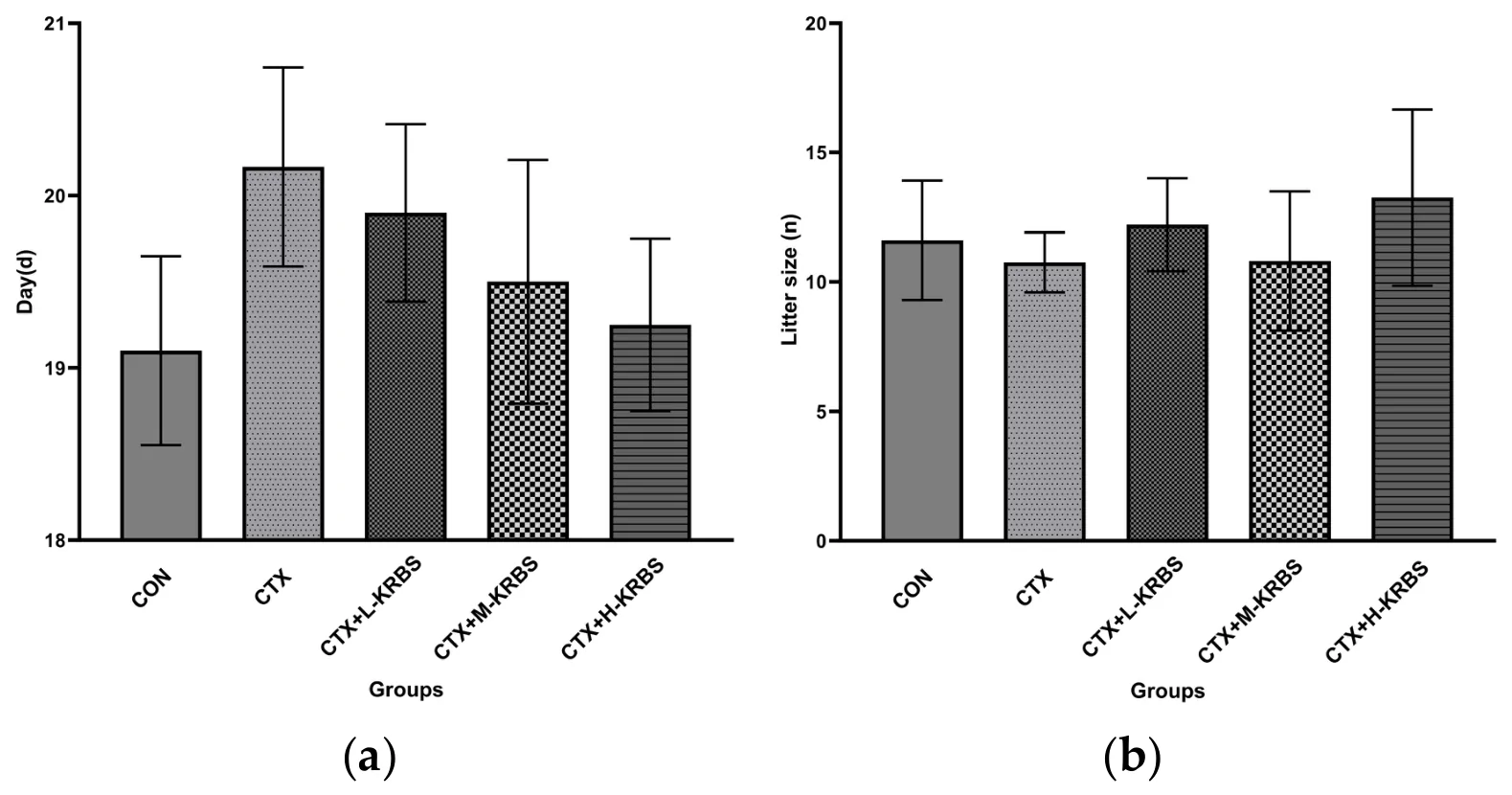

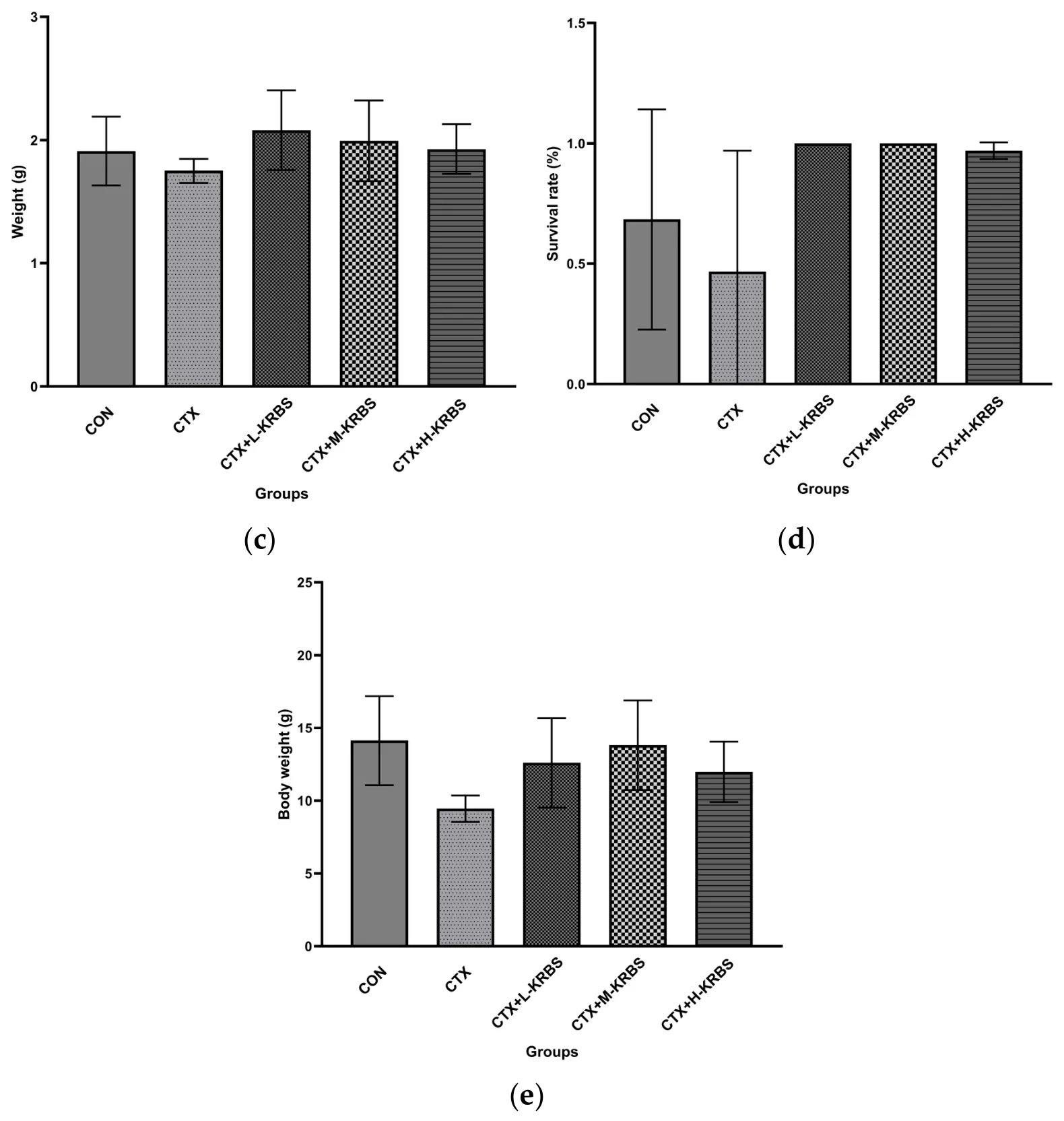
Effects of KRBS supplementation on pregnancy duration (**a**), litter size (**b**), average birth weight (**c**), offspring survival rate (**d**), and weaning weight (**e**) in CTX-induced ovarian dysfunction mice. Data are presented as mean ± SD (*n* = 10 per group). CON, normal control; CTX, cyclophosphamide; KRBS, Pueraria–black soybean compound powder; L-KRBS, low-dose KRBS; M-KRBS, medium-dose KRBS; H-KRBS, high-dose KRBS.

Reproductive performance assessment revealed that KRBS supplementation tended to improve gestation duration, with a dose-related trend across the three dose levels (Figure 4a). No significant differences were detected among groups in litter size or average birth weight (Figure 4b,c). The CTX group had a lower offspring survival rate, and the KRBS-supplemented groups showed a trend toward higher survival rates relative to both the CON and CTX groups; however, these differences were not statistically significant (Figure 4d, *p* > 0.05). A similar increasing trend in average weaning weight was observed following KRBS supplementation (Figure 4e). The lack of significant differences in most reproductive parameters may be due to the small sample size (*n* = 10 per group), the relatively short 8-week intervention, or the incomplete reversibility of CTX-induced ovarian damage.

### 3.5. Effects of KRBS on Gene Expression in Ovarian and Uterine Tissues

**Figure 5:**
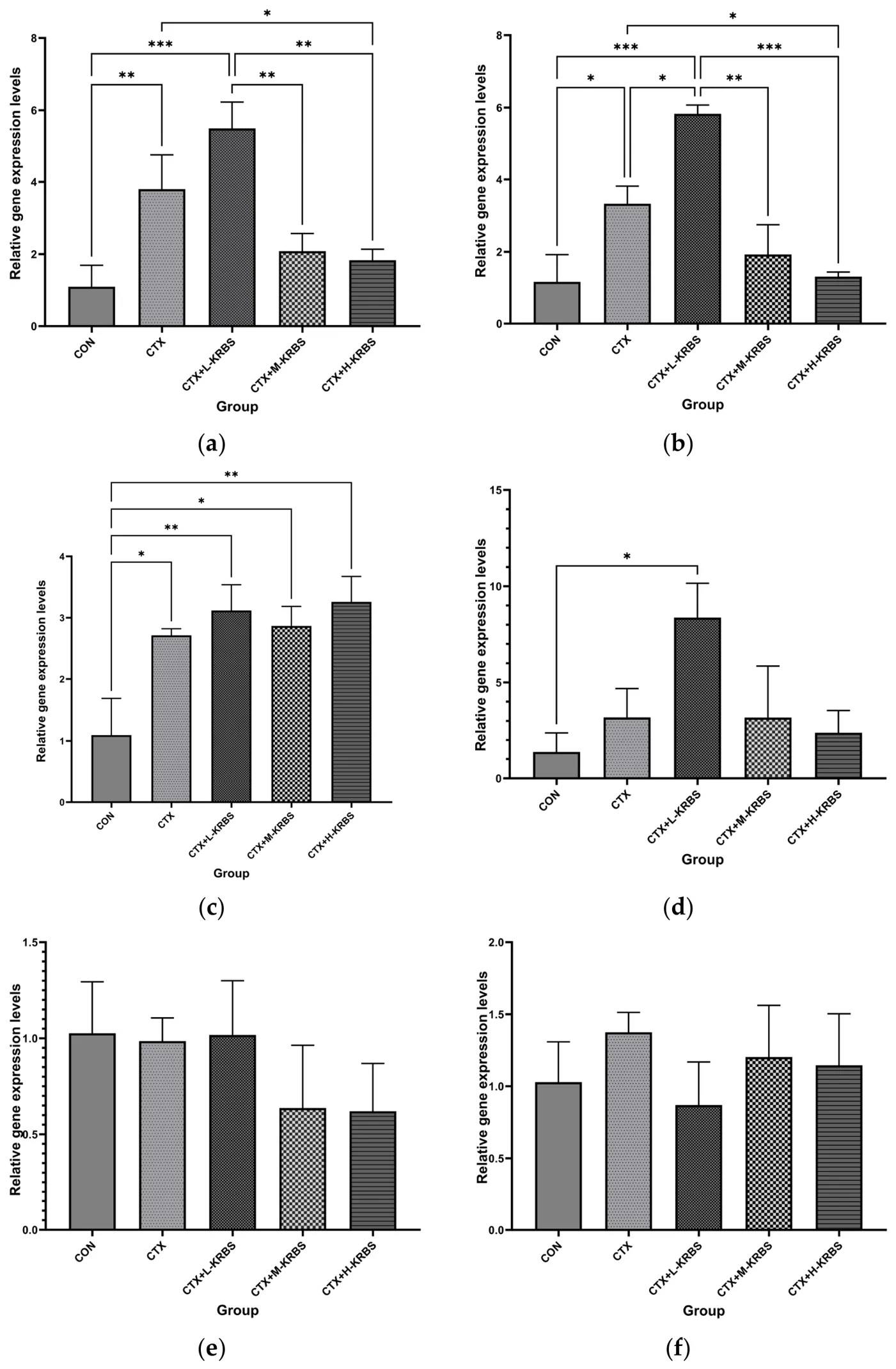
Effects of KRBS supplementation on the expression of reproduction-related genes in mice with CTX-induced ovarian dysfunction. (**a**–**c**) Expression of uterine genes: (**a**) *Esr1*, (**b**) *Vegfa*, and (**c**) *Col1a1*. (**d**–**f**) Expression of ovarian genes: (**d**) *Amh*, (**e**) *Bax*, and (**f**) *Bcl2*. Data are presented as mean ± SD (*n* = 10 per group). * *p* < 0.05, ** *p* < 0.01 vs. the CTX group; * *p* < 0.05, ** *p* < 0.01, *** *p* < 0.001 vs. the CON group. CON, normal control; CTX, cyclophosphamide; KRBS, Pueraria–black soybean compound powder; L-KRBS, low-dose KRBS; M-KRBS, medium-dose KRBS; H-KRBS, high-dose KRBS; *Esr1*, estrogen receptor α; *Vegfa*, vascular endothelial growth factor A; *Col1a1*, collagen type I alpha 1; *Amh*, anti-Müllerian hormone; *Bax*, Bcl-2-associated X protein; *Bcl2*, B-cell lymphoma 2.

The expression of reproduction-related genes in uterine tissues was examined (Figure 5a–c). *Esr1*, *Vegfa*, and *Col1a1* expression levels were significantly elevated in the CTX group compared with the CON group (*p* < 0.05). For *Esr1* (Figure 5a), the CTX+L-KRBS group showed a trend toward higher expression relative to the CTX group, although this difference was not statistically significant. In contrast, *Esr1* expression was lower in the CTX+M-KRBS and CTX+H-KRBS groups than in the CTX group, with a significant reduction in the CTX+H-KRBS group (*p* < 0.05) and expression levels approaching those of the CON group. *Vegfa* expression (Figure 5b) was significantly increased in the CTX+L-KRBS group compared with the CTX group (*p* < 0.05), while the CTX+M-KRBS and CTX+H-KRBS groups showed decreased *Vegfa* expression relative to the CTX group, reaching statistical significance in the CTX+H-KRBS group (*p* < 0.05), with levels approaching those of the CON group. *Col1a1* expression (Figure 5c), a marker associated with uterine extracellular matrix remodeling, was significantly elevated in the CTX group versus the CON group (*p* < 0.05). KRBS supplementation did not significantly alter *Col1a1* expression relative to the CTX group; however, *Col1a1* expression remained significantly higher in the CTX+L-KRBS, CTX+M-KRBS, and CTX+H-KRBS groups compared with the CON group (*p* < 0.01, *p* < 0.05, and *p* < 0.01, respectively).

In ovarian tissues, *Amh* was differentially expressed following KRBS supplementation (Figure 5d). *Amh* trended higher in the CTX group than in the CON group, without reaching statistical significance. Low-dose KRBS supplementation further enhanced *Amh* expression, with the CTX+L-KRBS group showing the highest expression level and reaching statistical significance (*p* < 0.05). In contrast, the CTX+M-KRBS and CTX+H-KRBS groups showed a trend toward lower *Amh* expression compared with the CTX+L-KRBS group, although these differences were not statistically significant. For the apoptosis-related gene *Bax* (Figure 5e), expression levels were broadly similar among the CON, CTX, and CTX+L-KRBS groups. The CTX+M-KRBS and CTX+H-KRBS groups showed a trend toward lower *Bax* expression relative to the CTX group, without reaching statistical significance. For *Bcl2* (Figure 5f), expression trended higher in the CTX group than in the CON group. The CTX+L-KRBS group trended lower, while the CTX+M-KRBS and CTX+H-KRBS groups trended higher relative to the CTX group; none of these differences reached statistical significance.

Together, these results indicate that KRBS supplementation altered the expression of apoptosis-related genes in ovarian tissue and genes involved in steroid responsiveness, angiogenesis, and extracellular matrix remodeling in uterine tissue.

## 4. Discussion

In modern livestock production, female reproductive efficiency is a major determinant of economic performance. Environmental stress, oxidative stress, and nutritional imbalances can impair ovarian function and compromise reproductive outcomes [20,21,22]. Developing safe and effective nutritional strategies to support female reproductive health has therefore become a priority in animal nutrition research. Plant-derived feed additives rich in flavonoids, polyphenols, and other bioactive compounds have been widely investigated because of their antioxidant properties and potential benefits for reproductive performance in livestock [23,24]. To evaluate the effects of plant-based dietary intervention on reproductive dysfunction, a cyclophosphamide (CTX)-induced ovarian dysfunction mouse model was employed in the present study. This model induces ovarian dysfunction characterized by disrupted estrous cycles, altered serum follicle-stimulating hormone (FSH) levels, and increased follicular atresia and has been widely used to evaluate whether dietary interventions can improve ovarian function [25].

In this study, KRBS supplementation partially alleviated CTX-induced reproductive dysfunction in mice, with the clearest changes seen in ovarian morphology and follicular dynamics. The improvement in ovarian index and the preservation of primordial follicles are consistent with what has been reported for puerarin and soy isoflavones, compounds that are known to help preserve follicular integrity in the setting of oxidative stress [26,27]. Uterine responses showed dose-dependent variation: the high dose promoted recovery, while the medium dose showed no improvement, suggesting that uterine sensitivity to phytoestrogens may depend on dose. KRBS also modulated reproductive endocrine parameters. The reduction in serum FSH toward control-group levels, particularly in the CTX+L-KRBS and CTX+H-KRBS groups, may reflect partial recovery of ovarian endocrine regulation associated with improved follicular status, rather than direct pharmacological suppression of FSH secretion. This interpretation is consistent with previous reports suggesting that phytoestrogens may influence hypothalamic–pituitary–gonadal axis regulation [28]. Serum LH trends, although not statistically significant, were directionally consistent with this model. Similarly, the favorable trends in offspring survival and weaning weight, while not reaching statistical significance within the constraints of this study, suggest that the improvements in ovarian and endocrine parameters warrant further investigation in adequately powered studies. These findings suggest that KRBS supports reproductive function through improvements in ovarian status and endocrine regulation, while uterine responses followed a more complex, dose-sensitive pattern.

In ovarian tissue, the gene expression profiles observed in this study provide mechanistic insight into how KRBS may preserve follicular reserve under CTX-induced stress. *Amh*, a widely used marker of ovarian follicular reserve status [29], was upregulated following KRBS supplementation, particularly in the CTX+L-KRBS group. Since *Amh* is mainly produced by growing follicles, its upregulation following KRBS supplementation is consistent with the histological evidence of primordial follicle preservation and suggests that KRBS may contribute to the maintenance of follicular development under ovarian stress. Concurrently, the shift in the *Bax*/*Bcl2* expression ratio—a trend toward decreased Bax and increased *Bcl2* in the CTX+M-KRBS and CTX+H-KRBS groups—points toward attenuation of the mitochondrial apoptotic pathway. This finding is mechanistically relevant: CTX induces granulosa cell apoptosis through oxidative DNA damage and subsequent activation of the intrinsic apoptotic cascade, a process regulated in part by the *Bax*/*Bcl2* balance. Plant-derived polyphenols and flavonoids, including puerarin and soy isoflavones, have been reported to preserve the *Bax*/*Bcl2* balance through antioxidant mechanisms [30,31]. These findings extend this pattern to a composite plant formulation, suggesting that the multi-component nature of KRBS may be associated with antioxidant-related and apoptosis-related pathways.

In the uterus, the coordinated downregulation of *Esr1* and *Vegfa* by high-dose KRBS, with expression levels returning toward those of the control group, suggests changes associated with endocrine status and vascular-related gene expression rather than reflecting broad transcriptional suppression. *Esr1* mediates uterine responsiveness to estrogen signaling [32,33], and *Vegfa* governs angiogenesis and vascular remodeling in reproductive tissues [34,35]; the parallel changes in these genes suggest that KRBS may influence uterine gene expression indirectly through modulation of endocrine status, consistent with the FSH and LH trends described above. In contrast, *Col1a1* remained persistently elevated across all KRBS-treated groups. As the principal collagen of the uterine ECM, *Col1a1* is upregulated during tissue injury and subsequent remodeling [36,37]. The persistent elevation of *Col1a1* following KRBS supplementation may indicate that ECM turnover proceeds on a longer timescale than hormonal and vascular recovery and may require extended intervention or additional matrix-targeted support. Together, these patterns suggest that systemic endocrine and vascular changes may contribute to KRBS-mediated uterine recovery, while structural remodeling of the ECM may occur on a slower timescale and represent a distinct aspect of tissue recovery.

Collectively, these findings indicate that KRBS has potential as a plant-based feed additive for supporting ovarian function and endocrine regulation.

Whether these findings translate to livestock production remains to be determined. Species differences in reproductive physiology, phytoestrogen metabolism, and dose–response relationships may influence KRBS efficacy, and doses established in mice cannot be directly extrapolated to economically important livestock such as sows, laying hens, and dairy cows. Practical deployment of KRBS also hinges on raw material standardization, production cost, bioactive compound stability during feed processing and storage, appropriate dietary inclusion rates, and long-term safety evaluation of phytoestrogen exposure in target species. This study has several limitations: the use of a single CTX-induced mouse model, an 8-week intervention period, the absence of oxidative stress marker data and intestinal microbiota–gonadal axis profiling, and the unavailability of KRBS formulation details for independent replication. Finally, the molecular findings are based solely on mRNA expression data: protein-level verification was not performed, and the mechanistic inferences should be interpreted with caution. Future livestock studies with longer feeding periods and broader outcome measures are warranted to evaluate the efficacy, safety, and feasibility of KRBS. These findings support the potential use of botanical combinations with complementary biological activities for developing phytogenic feed additives. This approach is consistent with the formulation principles of traditional Chinese veterinary medicine.

## 5. Conclusions

In conclusion, Pueraria–black soybean compound powder (KRBS) supplementation partially alleviated CTX-induced ovarian dysfunction in mice by improving ovarian morphology, supporting follicular development, and regulating reproductive endocrine status, with favorable trends in reproductive outcomes. The beneficial effects of KRBS may be associated with alterations in ovarian reserve-related responses, apoptosis-related gene expression, and uterine gene expression involved in tissue remodeling and vascular regulation. These findings provide preliminary evidence that KRBS may serve as a candidate plant-derived nutritional strategy for preserving ovarian function under chemotherapy-induced stress. Further validation in target livestock species is required to determine the optimal dosage, long-term safety, and practical feasibility of KRBS before its application in animal production.

## Figures and Tables

**Table 1:** Chemical characterization and nutritional composition of the Pueraria–black soybean compound powder (KRBS) per 100 g.

Component	Value
Energy	1588 kJ
Protein	11.5 g
Fat	2.2 g
Carbohydrate	77.1 g
Sodium	80 mg
Vitamin E	10.72 mg α-TE
Vitamin B_1_ (Thiamine)	1.68 mg
Vitamin B_6_	1.68 mg
Vitamin B_12_	5.00 μg
Vitamin C (Ascorbic acid)	220.0 mg
Folic acid	590 μg DFE

**Table 2:** Body weight (g) of mice before CTX administration, after CTX administration, and after the 8-week KRBS intervention.

Group	Before CTX	After CTX	After 8-Week KRBS
CON	29.79 ± 2.93	34.09 ± 2.34	48.92 ± 3.41 ^a^
CTX	29.26 ± 1.59	31.05 ± 3.13	43.56 ± 2.70 ^b^
CTX+L-KRBS	29.75 ± 3.64	31.74 ± 2.57	43.17 ± 3.75 ^b^
CTX+M-KRBS	30.03 ± 2.63	31.95 ± 2.55	43.87 ± 3.01 ^b^
CTX+H-KRBS	29.44 ± 2.64	32.00 ± 3.65	44.01 ± 2.89 ^c^

Data are presented as mean ± SD (*n* = 10 per group). ^a–c^; Values within the same column not sharing a common superscript differ significantly (^b^ vs. ^a^, *p* < 0.01; ^c^ vs. ^a^, *p* < 0.05). CON, normal control; CTX, cyclophosphamide; KRBS, Pueraria–black soybean compound powder; L-KRBS, low-dose KRBS; M-KRBS, medium-dose KRBS; H-KRBS, high-dose KRBS.

## Data Availability

The data presented in this study are available on request from the corresponding author.

## Appendix A

**Table A1:** The gene-specific primers for RT-qPCR analysis.

Genes	Forward (5′–3′)	Reverse (5′–3′)
Esr1	CTTGCTCTTGGACAGGGACA	ACTTGACGTAGCCAGCAACA
Vegfa	AAAACACAGACTCGCGTTGC	CCTCTCCCTTCATGTCAGGC
Col1a1	CCAGCCGCAAAGAGTCTACA	CAGGTTTCCACGTCTCACCA
Amh	CTGATTCCCGCTGTTTCACG	AGCGAGTGAGGGTCTCTAGG
Bax	TGCTACAGGGTTTCATCCAGG	TTCATCTCCAATTCGCCGGA
Bcl2	TCGAGAAGAGCAGCCCAATG	ACATCTCAAGCCTTCACGCA

Notes: All primers were synthesized by Sangon Biotech (Shanghai) Co., Ltd., Shanghai, China.
